# Supplementary material for: Interest in brief resistance training workouts among older US adults with and without mobility disability
Source: PLoS One. 2025 Aug 29;20(8):e0329684. doi: 10.1371/journal.pone.0329684 (PMC12396725; doi:10.1371/journal.pone.0329684)
Supplement: Attachment — Submitted filename: Response to Reviewers.docx [file pone.0329684.s002.docx]

Journal Requirements:

1. When submitting your revision, we need you to address these additional requirements.

Please ensure that your manuscript meets PLOS ONE's style requirements, including those for file naming. The PLOS ONE style templates can be found at

[https://journals.plos.org/plosone/s/file?id=wjVg/PLOSOne_formatting_sample_main_body.pdf](https://urldefense.com/v3/__https:/journals.plos.org/plosone/s/file?id=wjVg*PLOSOne_formatting_sample_main_body.pdf__;Lw!!Ls64Rlj6!3i4U7MiF4LjYNRNlGJGUn03xLRgEX8GXIUxpo4uc-KwjqlyrackgKFqBM8K_UpO6nmVSMhMoAjEqqXiUMbeTc3Ck$) and

[https://journals.plos.org/plosone/s/file?id=ba62/PLOSOne_formatting_sample_title_authors_affiliations.pdf](https://urldefense.com/v3/__https:/track.editorialmanager.com/CL0/https:*2F*2Fjournals.plos.org*2Fplosone*2Fs*2Ffile*3Fid=ba62*2FPLOSOne_formatting_sample_title_authors_affiliations.pdf/1/010f01953745dee2-d82fab83-5721-471f-8e2e-20a1ce7ffcb3-000000/diiQ4X86Qt6cD_7UeUctpW2HGprfsCiwBIl5x9akTJI=199__;JSUlJSUlJQ!!Ls64Rlj6!3i4U7MiF4LjYNRNlGJGUn03xLRgEX8GXIUxpo4uc-KwjqlyrackgKFqBM8K_UpO6nmVSMhMoAjEqqXiUMU6PN_ZH$)

Thank you, and sorry for the citation formatting oversight. I believe that the style requirements are now met.

2. Please provide additional details regarding participant consent. In the ethics statement in the Methods and online submission information, please ensure that you have specified (1) whether consent was informed and (2) what type you obtained (for instance, written or verbal, and if verbal, how it was documented and witnessed). If your study included minors, state whether you obtained consent from parents or guardians. If the need for consent was waived by the ethics committee, please include this information

Sorry for this oversight. The following statement has been added to the Methods section of the manuscript:

All participants were provided with a brief description of the study, and informed that their choosing to advance to the survey constituted agreement to participate in the study. The need for written consent was waived by the institutional review board, as data was collected anonymously.

3. Thank you for stating the following in the Competing Interests section: “Christopher Sciamanna has an investment, such as stock, in a company which has begun to investigate the possibility of creating a business that provides exercise programs. All other authors declare that they have no known competing financial interests or personal relationships that could have appeared to influence the work reported in this paper.”

Please confirm that this does not alter your adherence to all PLOS ONE policies on sharing data and materials, by including the following statement: "This does not alter our adherence to PLOS ONE policies on sharing data and materials.” (as detailed online in our guide for authors [http://journals.plos.org/plosone/s/competing-interests](https://urldefense.com/v3/__http:/journals.plos.org/plosone/s/competing-interests__;!!Ls64Rlj6!3i4U7MiF4LjYNRNlGJGUn03xLRgEX8GXIUxpo4uc-KwjqlyrackgKFqBM8K_UpO6nmVSMhMoAjEqqXiUMeSR9qkY$)). If there are restrictions on sharing of data and/or materials, please state these. Please note that we cannot proceed with consideration of your article until this information has been declared.

Please include your updated Competing Interests statement in your cover letter; we will change the online submission form on your behalf.

Thank you. The following statement has been added to the cover letter:

One of the co-authors, Christopher Sciamanna, has an investment, such as stock, in a company which has begun to investigate the possibility of creating a business that provides exercise programs. All other authors declare that they have no known competing financial interests or personal relationships that could have appeared to influence the work reported in this paper. This does not alter our adherence to PLOS One policies on sharing data and materials.

4. We noted in your submission details that a portion of your manuscript may have been presented or published elsewhere. [Data related to this publication have been published previously in PLOS One. The published manuscript has been provided with this submission.] Please clarify whether this publication was peer-reviewed and formally published. If this work was previously peer-reviewed and published, in the cover letter please provide the reason that this work does not constitute dual publication and should be included in the current manuscript.

The following paragraph is now included in the cover letter:

This research is related to a manuscript previously published in PLOS One. The related manuscript is focused on evaluating the equivalence of preferences and self-efficacy ratings, as well as identifying the possible implications of using either preference or self-efficacy ratings to design strength training programs for older adults to enhance long-term maintenance. In contrast, this research identifies patterns of health issues/comorbidities and predictors that influence strength training program preference. Therefore, this work does not constitute dual publication. The related manuscript published in PLOS One has been included with this submission.

5. When completing the data availability statement of the submission form, you indicated that you will make your data available on acceptance. We strongly recommend all authors decide on a data sharing plan before acceptance, as the process can be lengthy and hold up publication timelines. Please note that, though access restrictions are acceptable now, your entire data will need to be made freely accessible if your manuscript is accepted for publication. This policy applies to all data except where public deposition would breach compliance with the protocol approved by your research ethics board. If you are unable to adhere to our open data policy, please kindly revise your statement to explain your reasoning and we will seek the editor's input on an exemption. Please be assured that, once you have provided your new statement, the assessment of your exemption will not hold up the peer review process.

Thank you for highlighting this. We have an established methodology of hosting data for availability related to publications within Penn State University. The timeline for this process is typically a matter of days.

6. Please include captions for your Supporting Information files at the end of your manuscript, and update any in-text citations to match accordingly. Please see our Supporting Information guidelines for more information: [http://journals.plos.org/plosone/s/supporting-information](https://urldefense.com/v3/__http:/journals.plos.org/plosone/s/supporting-information__;!!Ls64Rlj6!3i4U7MiF4LjYNRNlGJGUn03xLRgEX8GXIUxpo4uc-KwjqlyrackgKFqBM8K_UpO6nmVSMhMoAjEqqXiUMfAT8jWd$).

Thank you. This change has been made.

Editorial Recommendation

Strengthen the discussion by acknowledging the limitations of self-reported preferences versus actual exercise behavior and their implications for generalizability.

Good point. The following statement is now included in the discussion section:

Second, the survey only asked about preference, not intent, specific plans for change, or actual exercise behavior, so it remains unknown whether these stated preferences would lead more older adults to actually participate in shorter programs if offered, or continue them longer if they began. In a previous study, more than 50% of older adults expressed an interest in a free strength training program, yet fewer than 25% of older adults with access to free programs that include strength training actually participate.

Provide more details on sample representativeness and potential biases, including how quota sampling was enforced and addressing self-selection bias due to digital survey recruitment.

Thank you for highlighting this issue. The following has been added in the participants section:

Demographic information was collected prior to completing the rest of the survey; once quotas were met for a particular demographic combination, no additional survey completions were allowed by people in that particular demographic.

The paragraph below also appears in the discussion section:

While the study was conducted with a national sample in a commonly used survey panel, and recruited a diverse sample that was representative of the US population over the age of 65, the conclusions must be judged with several limitations in mind. First, the sample was of individuals with Internet access while only 75% of older adults report access to the Internet. A nationwide survey using the same Qualtrics panel in 2020 observed greater rates of college completion (55.8%) than observed by the Census Bureau (37.9%), suggesting that the sampling method used by Qualtrics may be biased towards respondents that are more educated than the general public.

Discuss the feasibility and long-term sustainability of a 5-minute RT program, considering factors such as program adherence, supervision, and real-world implementation.

Thank you for this suggestion. The followings section has been added to the discussion section:

It remains unclear if long-term participation (i.e., adherence) would differ between short- and longer-session RT programs. However, the benefit of increased uptake of short RT programs is of significant potential public health benefit, bringing the potential for feasible RT programs to the populations that would benefit from them the most (i.e., worse self-reported health, more comorbidities, higher BMI, more use of mobility devices, more walking difficulty, and more falls in the past year). This format may also be particularly amenable to rehabilitation settings, where uptake and early adherence of exercise interventions is typically low.

Clarify how missing data were handled in the analysis and whether any sensitivity analyses were conducted to assess potential bias.

Thank you for this clarification. There was not missing data in this analysis, as part of the Qualtrics quota system requires a completed survey to meet the quota numbers. This has been clarified in the participant profile section:

In April 2022, 1162 adults began the survey. Participants that did not reach the end of the survey (n = 438), whose demographic quotas were filled before they completed their survey (n = 95), that completed the survey too quickly (n = 9), that failed the attention check (n = 5), or that were identified as bots using embedded survey fields (n = 4) were removed. Therefore, the final sample size included 611 participants who completed all questions needed for this analysis.

Address the lack of blinding in survey responses and discuss any measures taken to minimize social desirability bias.

Thank you for raising this issue. The survey responses were not conditional by participant, so there was no blinding that could occur.

The following has been added to the discussion section to reflect actions taken to minimize social desirability bias:

While the study was conducted with a national sample in a commonly used survey panel, and recruited a diverse sample that was representative of the US population over the age of 65 without requiring in-person data collection, the conclusions must be judged with several limitations in mind.

Enhance the discussion on the practical implications of shorter RT programs, particularly for public health interventions and rehabilitation settings.

Thank you for this suggestion. The followings section has been added to the discussion section:

It remains unclear if long-term participation (i.e., adherence) would differ between short- and longer-session RT programs. However, the benefit of increased uptake of short RT programs is of significant potential public health benefit, bringing the potential for feasible RT programs to the populations that would benefit from them the most (i.e., worse self-reported health, more comorbidities, higher BMI, more use of mobility devices, more walking difficulty, and more falls in the past year).

Consider adding a follow-up study examining actual adherence, physical function outcomes, and health benefits of a 5-minute RT program compared to traditional RT regimens.

We could not agree more. We are currently conducting a long-term randomized controlled trial of exactly this. The following statement has been added to the discussion.

Future studies should examine both adherence and outcome impacts of short RT programs compared to longer, traditional RT programs.

We appreciate your contributions to this field and look forward to your revised manuscript. Please submit your revisions along with a point-by-point response addressing these concerns.

Thank you very much for your time and effort to improve this manuscript. We appreciate your perspectives and insight.
